# Comparative study of the antioxidant and reactive oxygen species scavenging properties in the extracts of the fruits of *Terminalia chebula*, *Terminalia belerica *and *Emblica officinalis*

**DOI:** 10.1186/1472-6882-10-20

**Published:** 2010-05-13

**Authors:** Bibhabasu Hazra, Rhitajit Sarkar, Santanu Biswas, Nripendranath Mandal

**Affiliations:** 1 Division of Molecular Medicine, Bose Institute, P-1/12 CIT Scheme VIIM, Kolkata-700054, India

## Abstract

**Background:**

Cellular damage caused by reactive oxygen species (ROS) has been implicated in several diseases, and hence natural antioxidants have significant importance in human health. The present study was carried out to evaluate the *in vitro *antioxidant and reactive oxygen species scavenging activities of *Terminalia chebula*, *Terminalia belerica *and *Emblica officinalis *fruit extracts.

**Methods:**

The 70% methanol extracts were studied for *in vitro *total antioxidant activity along with phenolic and flavonoid contents and reducing power. Scavenging ability of the extracts for radicals like DPPH, hydroxyl, superoxide, nitric oxide, hydrogen peroxide, peroxynitrite, singlet oxygen, hypochlorous acid were also performed to determine the potential of the extracts.

**Results:**

The ability of the extracts of the fruits in exhibiting their antioxative properties follow the order *T. chebula *>*E. officinalis *>*T. belerica*. The same order is followed in their flavonoid content, whereas in case of phenolic content it becomes *E. officinalis *>*T. belerica *>*T. chebula*. In the studies of free radicals' scavenging, where the activities of the plant extracts were inversely proportional to their IC_50 _values, *T. chebula *and *E. officinalis *were found to be taking leading role with the orders of *T. chebula *>*E. officinalis *>*T. belerica *for superoxide and nitric oxide, and *E. officinalis *>*T. belerica *>*T. chebula *for DPPH and peroxynitrite radicals. Miscellaneous results were observed in the scavenging of other radicals by the plant extracts, viz., *T. chebula *>*T. belerica *>*E. officinalis *for hydroxyl, *T. belerica *>*T. chebula *>*E. officinalis *for singlet oxygen and *T. belerica *>*E. officinalis *>*T. chebula *for hypochlorous acid. In a whole, the studied fruit extracts showed quite good efficacy in their antioxidant and radical scavenging abilities, compared to the standards.

**Conclusions:**

The evidences as can be concluded from the study of the 70% methanol extract of the fruits of *Terminalia chebula*, *Terminalia belerica *and *Emblica officinalis*, imposes the fact that they might be useful as potent sources of natural antioxidant.

## Background

Oxidative stress plays an important role in the pathogenesis of various diseases such as atherosclerosis, alcoholic liver cirrhosis and cancer etc. Oxidative stress is initiated by reactive oxygen species (ROS), such as superoxide anion (O^-2^), perhydroxy radical (HOO-) and hydroxyl radical (HO·). These radicals are formed by a one electron reduction process of molecular oxygen (O_2_). ROS can easily initiate the lipid peroxidation of the membrane lipids, causing damage of the cell membrane of phospholipids, lipoprotein by propagating a chain reaction cycle [1,2]. Thus, antioxidants defense systems have coevolved with aerobic metabolism to counteract oxidative damage from ROS. Most living species have efficient defense systems to prevent themselves against oxidative stress induced by ROS [3]. Recent investigations have shown that the antioxidant properties of plants could be correlated with oxidative stress defense and different human diseases and aging process etc [4]. In this respect flavonoids and other polyphenolic compounds have received the greatest attention.

The fruits of *Terminalia chebula *Retz, *Terminalia belerica *Roxb, and *Emblica officinalis *Gaertn are widely used in the Indian traditional system of medicine [5]. The half ripe fruit of *T. belerica *and the pericarp of *T. chebula *fruit were reported to be purgative [5]. The fruit of *T. chebula *was traditionally used to cure asthma, urinary disorders, heart disease and it has cardiotonic activity [6,7]. In Ayurveda, the fruit of *E. officinalis *is used as a cardiotonic, cerebral and intestinal tonic [8], and it is also reported to have anticancer properties [9,8]. The fruit of *E. officinalis *is a rich source of vitamin C, a well-known antioxidant [10]. The crude extract of *E. officinalis *was reported to counteract the hepatotoxic and renotoxic effects of metals [11] due to antioxidant properties.

This present study is aimed to assess the antioxidant capacity of the 70% methanol extracts of *T. chebula*, *T. belerica *and *E. officinalis *fruits, through their measurement of activities in scavenging of different free radicals including hydroxyl, superoxide, nitric oxide, hydrogen peroxide, peroxynitrite, singlet oxygen, hypochlorous acid, phenol, flavonoid and ascorbic acid content and total antioxidant activity with ABTS and DPPH.

## Methods

### Chemicals

2,2'-azinobis-(3-ethylbenzothiazoline-6-sulfonic acid) (ABTS) was obtained from Roche diagnostics, Mannheim, Germany. 6-hydroxy-2,5,7,8-tetramethylchroman-2-carboxylic acid (Trolox) was obtained from Fluka, Buchs, Switzerland. Potassium persulfate (K_2_S_2_O_8_), 2-deoxy-2-ribose, mannitol, sodium nitroprusside (SNP), lipoic acid 5,5'-dithiobis-2-nitrobenzoic acid (DTNB), 1-chloro-2,4-dinitrobenzene (CDNB), glutathione reduced and quercetin were obtained from Sisco Research Laboratories Pvt. Ltd, Mumbai, India. Folin-ciocalteu reagent, xylenol orange and *N*, *N*-dimethyl-4-nitrosoaniline were obtained from Merck, Mumbai, India. Gallic acid, 1,1-Diphenyl-2-Picrylhydrazyl (DPPH) and curcumin were obtained from MP Biomedicals, France. Catalase was obtained from HiMedia Laboratories Pvt. Ltd, Mumbai, India. Evans blue was purchased from BDH, England. Diethylene-triamine-pentaacetic acid (DTPA) was obtained from Spectrochem Pvt. Ltd, Mumbai, India. Thiobarbituric acid (TBA) was obtained from Loba Chemie, Mumbai, India.

### Plant material

The fruits of *T. chebula*, *T. belerica *and *E. officinalis *were collected from Bankura district of West Bengal, India. The plant was identified by the Central Research Institute (Ayurveda), Kolkata, India, where specimens of each plant were deposited (Table 1).

**Table 1:** Voucher specimen number of three plants

**Sl. No**.	Plant	**Specimen No**.
1.	*Terminalia chebula*	CRHS 113/08
2.	*Terminalia belerica*	CRHS 114/08
3.	*Emblica officinalis*	CRHS 115/08

### Animals

Male Swiss albino mice (20 ± 2 g) were purchased from Chittaranjan National Cancer Institute (CNCI), Kolkata, India and were maintained under a constant 12-h dark/light cycle at an environmental temperature of 22 ± 2°C. The animals were fed with normal laboratory pellet diet and water ad libitum. The institutional animal ethics committee approved all experimental procedure.

### Extraction

The powder (100 g) of the individual normal air-dried fruits of *T. chebula*, *T. belerica *and *E. officinalis *were stirred using a magnetic stirrer with a 7:3 mixture of methanol: water (500 ml) for 15 hours; the mixture was then centrifuged at 2850 × *g *and the supernatant decanted. The process was repeated by adding the solvent with the precipitated pellet. The supernatants were collected, concentrated in a rotary evaporator [250-200 mbar at 37°C] and lyophilized. The yields for the plants materials were 5.6 g, 3.7 g and 4.2 g for *T. chebula*, *T. belerica *and *E. officinalis*, respectively. The dried extracts were stored at -20°C until use.

### *In vitro *Antioxidant assay

#### Total antioxidant activity

Antioxidant capacity was measured based on the scavenging of ABTS^.^^+ ^radical cation by the sample in comparison to trolox standard [12]. ABTS solution was mixed with potassium persulfate and incubated for 12-16 h in dark to generate ABTS^.^^+ ^radical cation. Then 10 μl sample solution was mixed with 1 ml ABTS^.^^+ ^solution and the absorbance was measured at λ = 734 nm. All experiments were repeated six times. The percentage inhibition of absorbance was calculated and plotted as a function of concentration of standard and sample to determine the trolox equivalent antioxidant concentration (TEAC). To calculate the TEAC, the gradient of the plot for the sample was divided by the gradient of the plot for trolox.

#### DPPH radical scavenging assay

The complementary study for the antioxidant capacity of the fruit extract was confirmed by the DPPH scavenging assay according to Mahakunakorn et al. [13], with slight modification. Different concentrations (0-100 μg/ml) of the extracts and the standard trolox were mixed with equal volume of ethanol. Then 50 μl of DPPH solution (1 mM) was pipetted into the previous mixture and stirred thoroughly. The resulting solution was kept standing for 2 minutes before the optical density (OD) was measured at λ = 517 nm. The measurement was repeated with six sets. The percentage radical scavenging activity was calculated from the following formula:

Where A_0 _was the absorbance of the control and A_1 _was the absorbance in the presence of the samples and standard.

#### Hydroxyl radical scavenging assay

The scavenging assay for hydroxyl radical was performed by a standard method [12]. Hydroxyl radical was generated by the Fenton reaction using a Fe^3+^-ascorbate-EDTA-H_2_O_2 _system. The assay quantifies the 2-deoxyribose degradation product, by its condensation with TBA. All tests were carried out six times. Mannitol, a classical. OH scavenger, was used as a standard compound. Percent inhibition was evaluated by the following equation:

Where A_0 _was the absorbance of the control and A_1 _was the absorbance in the presence of the samples and standard.

#### Superoxide radical scavenging assay

Measurements of superoxide anion scavenging activities of the samples and standard quercetin were done based on the reduction of NBT according to a previously described method [12]. Superoxide radical is generated by a non-enzymatic system of phenazine methosulfate-nicotinamide adenine dinucleotide (PMS/NADH). These radicals reduce nitro blue tetrazolium (NBT) into a purple colored formazan which was measured spectrophotometrically at λ = 562 nm. All tests were performed six times. The percentage inhibition of superoxide anion generation was calculated using the following formula:

Where A_0 _was the absorbance of the control and A_1 _was the absorbance in the presence of the samples and standard.

#### Nitric oxide radical scavenging assay

Sodium nitroprusside (SNP) gives rise to nitric oxide that under interaction with oxygen produce nitrite ions measured by Griess Illosvoy reaction [12]. The chromophore generated was spectrophotometrically measured at λ = 540 nm against blank sample. All tests were performed six times. Curcumin was used as a standard. The percentage inhibition of nitric oxide radical generation was calculated using the following formula:

Where A_0 _was the absorbance of the control and A_1 _was the absorbance in the presence of the samples and standard.

#### Hydrogen peroxide scavenging assay

FOX-reagent method was used to determine this activity of the sample and the reference compound sodium pyruvate, as previously described [12]. The absorbance of the ferric-xylenol orange complex was measured at λ = 560 nm. All tests were carried out six times. The percentage of scavenging of hydrogen peroxide of fruit extracts and standard compound:

where A_0 _was the absorbance of the control, and A_1 _was the absorbance in the presence of the sample of fruit extracts and standard.

#### Peroxynitrite scavenging assay

Peroxynitrite (ONOO^-^) synthesis was done 12 hrs before the assay, according to Beckman et al [14]. Acidic solution (0.6 M HCl) of 5 ml H_2_O_2 _(0.7 M) was mixed with 5 ml of 0.6 M KNO_2 _on an ice bath for 1 s and 5 ml of ice-cold 1.2 M NaOH was added to the reaction mixture. Excess H_2_O_2 _was adsorbed by granular MnO_2 _and the reaction mixture was left at -20°C. The concentration of the peroxynitrite solution was measured spectrophotometrically at λ = 302 nm (ε = 1670 M^-1 ^cm^-1^).

Evans blue bleaching assay was used to measure the peroxynitrite scavenging activity [12]. The percentage of scavenging of ONOO^- ^was calculated by comparing the results of the test and blank samples. All tests were performed six times. Gallic acid was used as reference compound. The percentage of scavenging of peroxynitrite anion was calculated using the following equation:

where A_0 _was the absorbance of the control, and A_1 _was the absorbance in the presence of the sample of fruit extracts and standard.

#### Singlet oxygen scavenging assay

Singlet oxygen (^1^O_2_) production, and at the same time, its scavenging by the samples and the reference compound lipoic acid can be monitored by *N*, *N*-dimethyl-4-nitrosoaniline (RNO) bleaching, using a earlier reported method [12]. Singlet oxygen was generated by a reaction between NaOCl and H_2_O_2 _and the bleaching of RNO was read at λ = 440 nm. All tests were performed six times. Singlet oxygen scavenging was calculated by the following formula:

where A_0 _was the absorbance of the control, and A_1 _was the absorbance in the presence of the sample of fruit extracts and standard.

### Hypochlorous acid scavenging assay

According to a previously described method [12], hypochlorous acid (HOCl) was prepared just before the experiment by adjusting the pH of a 10% (v/v) solution of NaOCl to pH 6.2 with 0.6 M H_2_SO_4 _and the concentration of HOCl was determined by taking the absorbance at λ = 235 nm using the molar extinction coefficient of 100 M^-1 ^cm^-1^. The scavenging activities of the fruit extracts and the standard, ascorbic acid, a potent HOCl scavenger was evaluated by measuring the decrease in the absorbance of catalase at λ = 404 nm. All tests were performed six times. The percentage of scavenging of HOCl was calculated using the following equation:

where A_0 _was the absorbance of the control, and A_1 _was the absorbance in the presence of the sample of fruit extracts and standard.

#### Reducing power assay

The Fe^3+^-reducing power of the extract was determined by a standard method [12]. In a phosphate buffer solution (0.2 M, pH 6.6), different concentrations (0.0-0.4 mg/ml) of the extract were mixed with potassium hexacyanoferrate (0.1%), followed by incubation. After incubation, the upper portion of the solution was diluted, and FeCl_3 _solution (0.01%) was added. The reaction mixture was left for 10 min at room temperature for colour development and the absorbance was measured at λ = 700 nm. All tests were performed six times. A higher absorbance of the reaction mixture indicated greater reducing power. Ascorbic acid was used as a positive control.

### Determination of total phenolic content

The amount of total phenolics present in the fruit extract was determined using Folin-Ciocalteu (FC) reagent by a formerly reported method [12]. A gallic acid standard curve (*R*^2 ^= 0.9468) was used to measure the phenolic content.

### Determination of total flavonoid content

The amount of total flavonoids was determined with aluminium chloride (AlCl_3_) according to a known method [12]. The flavonoid content was calculated from quercetin standard curve (*R*^2 ^= 0.9947).

### Determination of ascorbic acid content

The amount of total ascorbic acid was determined with ferroin complexation method with minor modification [15]. To 1 ml solution of the sample, 100 μl of 3.3*10^-3 ^M Iron (III)-phen colour reagent was added and the pH was adjusted to 4.5 with 20% sodium acetate. The absorbance was measured at λ = 512 nm and subsequently the ascorbate content was calculated from ascorbic acid standard curve (*R*^2 ^= 0.9554).

### *In vivo *Antioxidant assay

#### Experimental design

Mice were randomly divided into ten groups containing six animals in each group. Group I animals served as control and administered a single daily dose of normal saline. Groups II, III and IV received the *T. chebula *extract at a dose of 10, 50 and 100 mg/kg body weight, respectively. Groups V, VI and VII were given *T. belerica *extract at a dose of 10, 50 and 100 mg/kg body weight, respectively. The other three groups (Group VIII, IX and X) were administered *E. officinalis *extract at the same dose. The treatments were carried out orally for 7 days and on the 8th day all the animals were sacrificed by cervical dislocation. The liver was immediately removed and after washing with ice-cold saline it was homogenized in 10 volume of 0.1 M phosphate buffer (pH 7.4) containing 5 mM EDTA and 0.15 M NaCl, and centrifuged at 8000 g for 30 min at 4°C. The supernatant was collected and used for the assay of enzyme activities. Protein concentration was estimated according to Lowry method [16] using BSA as standard.

#### Assay of Antioxidant Enzymes

Superoxide dismutase (SOD) was assayed by measuring the inhibition of the formation of blue colored formazan at 560 nm according to the technique of Kakkar et al. [17]. Catalase (CAT) activity was measured by following the decrease in H_2_O_2 _concentration spectrophotometrically over time at 240 nm according to a previously described method [18]. Glutathione-S-transferase (GST) was determined by the method of Habig and Jacoby [19] based on the formation of GSH-CDNB conjugate and increase in the absorbance at 340 nm. Reduced glutathione (GSH) level was measured spectrophotometrically at 412 nm by the method of Ellman [20].

### Statistical analysis

All data were reported as the mean ± SD of six measurements. The statistical analysis was performed by KyPlot version 2.0 beta 15 (32 bit). The IC_50 _values were calculated by the formula, Y = 100*A1/(X + A1) where A1 = IC_50_, Y = response (Y = 100% when X = 0), X = inhibitory concentration. The IC_50 _values were compared by paired t test (two-sided). *p *< 0.05 was considered significant.

## Results

### Total antioxidant activity

**Figure 1:**
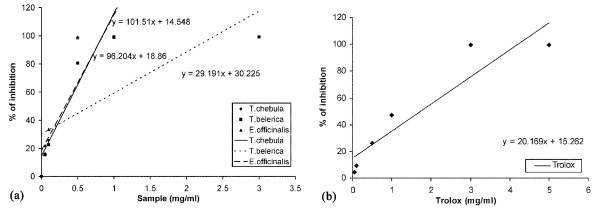
**Total antioxidant activity**. Total antioxidant activity of plant extract and trolox. Effect of (a) *T. chebula*, *T. belerica *and *E. officinalis *extracts and (b) reference compound trolox on ABTS radical cation decolorization assay. The percentage of inhibition was plotted against concentration of sample. All data are expressed as mean ± S.D. (n = 6).

The total antioxidant capacity of the extract was calculated from the decolorization of ABTS^**.+**^, upon interaction with the extract or standard trolox that suppressed the absorbance of the ABTS ^**.+ **^radical cation and the results, expressed as percentage inhibition of absorbance, are shown in Figure 1(a) and 1(b), respectively. The TEAC value of the extracts of *T. chebula*, *T. belerica *and *E. officinalis *were 4.52 ± 0.12, 1.01 ± 0.03 and 4.10 ± 0.17, respectively.

### DPPH Scavenging Activity

**Figure 2:**
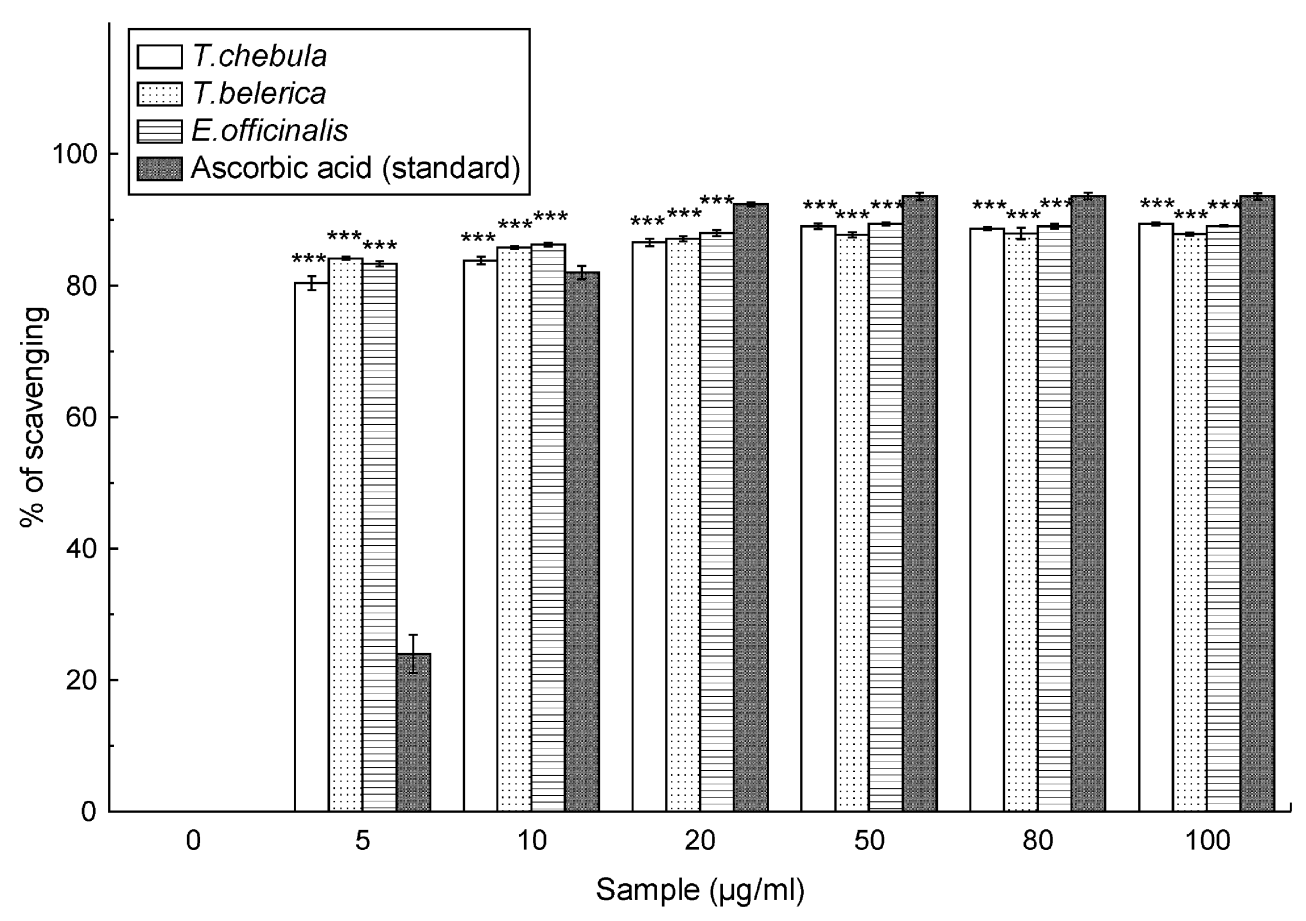
**DPPH scavenging activity**. Effect of the *T. chebula*, *T. belerica *and *E. officinalis *extracts and standard ascorbic acid on DPPH radical scavenging study. The data is expressed as % scavenging of DPPH radicals. The results are mean ± S.D. of six parallel measurements. ***p < 0.001 vs 0 μg/ml.

As is evident in the Figure 2 and the IC_50 _value of the samples, following the order *T. chebula, T. belerica *&*E. officinalis *(1.73 ± 0.07 μg/ml, 1.45 ± 0.02 μg/ml & 1.43 ± 0.03 μg/ml) in comparison to the ascorbic acid (5.29 ± 0.28 μg/ml) standard (Table 2) to scavenge the radical, it can be put forward as a fact that the extracts truly work as antioxidant.

**Table 2:** Comparison of the antioxidant and free radical scavenging capacities of 70% methanolic crudes of *Terminalia chebula*, *Terminalia belerica *and *Emblica officinalis*

Name of Assay	70% methanolic crudes of			Standard	Values of Standard compounds
			
	*Terminalia chebula*	*Terminalia belerica*	*Emblica officinalis*		
TEAC Values	4.52 ± 0.12	1.01 ± 0.03	4.10 ± 0.17	--	--
† Phenolic content	127.60 ± 0.001	133.00 ± 0.003	215.60 ± 0.004	--	--
‡ Flavonoid content	219.30 ± 0.01	138.30 ± 0.01	176.00 ± 0.01	--	--
§ Ascorbic acid content	46.74 ± 1.10	45.25 ± 0.75	71.08 ± 1.63	--	--

♣ IC_50 _values of the extracts for free radical scavenging capacity for:					

DPPH	1.73 ± 0.07***	1.45 ± 0.02***	1.43 ± 0.03***	Ascorbic acid	5.29 ± 0.28
Hydroxyl (OH^.^)	72.02 ± 8.99***	203.25 ± 1.87**	382.02 ± 7.88***	Mannitol	571.45 ± 20.12
Superoxide (O_2_^.-^)	13.42 ± 0.22***	18.18 ± 1.39***	13.82 ± 0.19***	Quercetin	42.06 ± 1.35
Nitric oxide (NO^.^)	33.28 ± 4.56***	40.83 ± 4.40***	33.89 ± 2.94***	Curcumin	90.82 ± 4.75
Peroxynitrite (ONOO^.-^)	1.27 ± 0.07***	0.99 ± 0.10**	0.83 ± 0.03^NS^	Gallic acid	0.88 ± 0.06
Singlet oxygen (^1^O_2_)	424.50 ± 24.70***	233.12 ± 48.68***	490.42 ± 159.59**	Lipoic acid	46.15 ± 1.16
Hypochlorous acid (HOCl)	433.60 ± 15.45**	271.51 ± 13.70**	420.58 ± 31.97***	Ascorbic acid	235.96 ± 5.75

† Phenolic content (mg/ml Gallic acid equivalent per 100 mg plant extract)

‡ Flavonoid content (mg/ml Quercetin equivalent per 100 mg plant extract)

§ Ascorbic acid content (mg/ml Ascorbic acid per 100 mg plant extract)

♣ IC_50 _Values are in μg/ml (except for Peroxynitrite scavenging assay where values are expressed in mg/ml). Each value represents mean ± S.D. (n = 6)

* p < 0.05; ** p < 0.01;*** p < 0.001; NS = Non significant

### Hydroxyl radical scavenging assay

**Figure 3:**
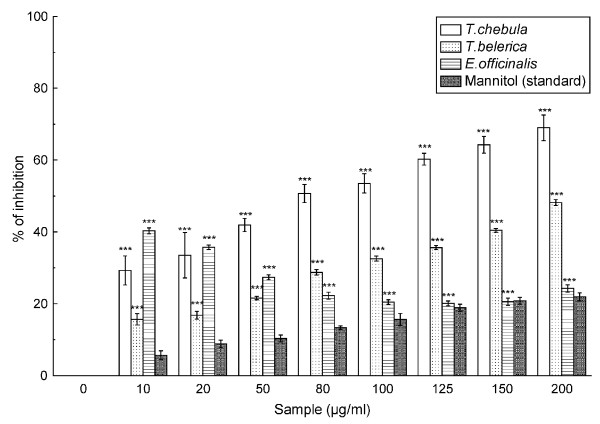
**Hydroxyl radical scavenging assay**. Hydroxyl radical scavenging activity of the *T. chebula*, *T. belerica *and *E. officinalis *extracts and the reference compound mannitol. The data represent the percentage of inhibition of deoxyribose degradation. The results are mean ± S.D. of six parallel measurements. ***p < 0.001 vs 0 μg/ml.

This assay shows the abilities of the extracts and standard mannitol to scavenge hydroxyl radical, as shown in Figure 3. The IC_50 _values (Table 2) of the extracts (in the order *T. chebula*, *T. belerica *and *E. officinalis*) and standard in this assay were 72.02 ± 8.99 μg/ml, 203.25 ± 1.87 μg/ml, 382.02 ± 7.88 μg/ml and 571.45 ± 20.12 μg/ml, respectively.

### Superoxide radical scavenging assay

**Figure 4:**
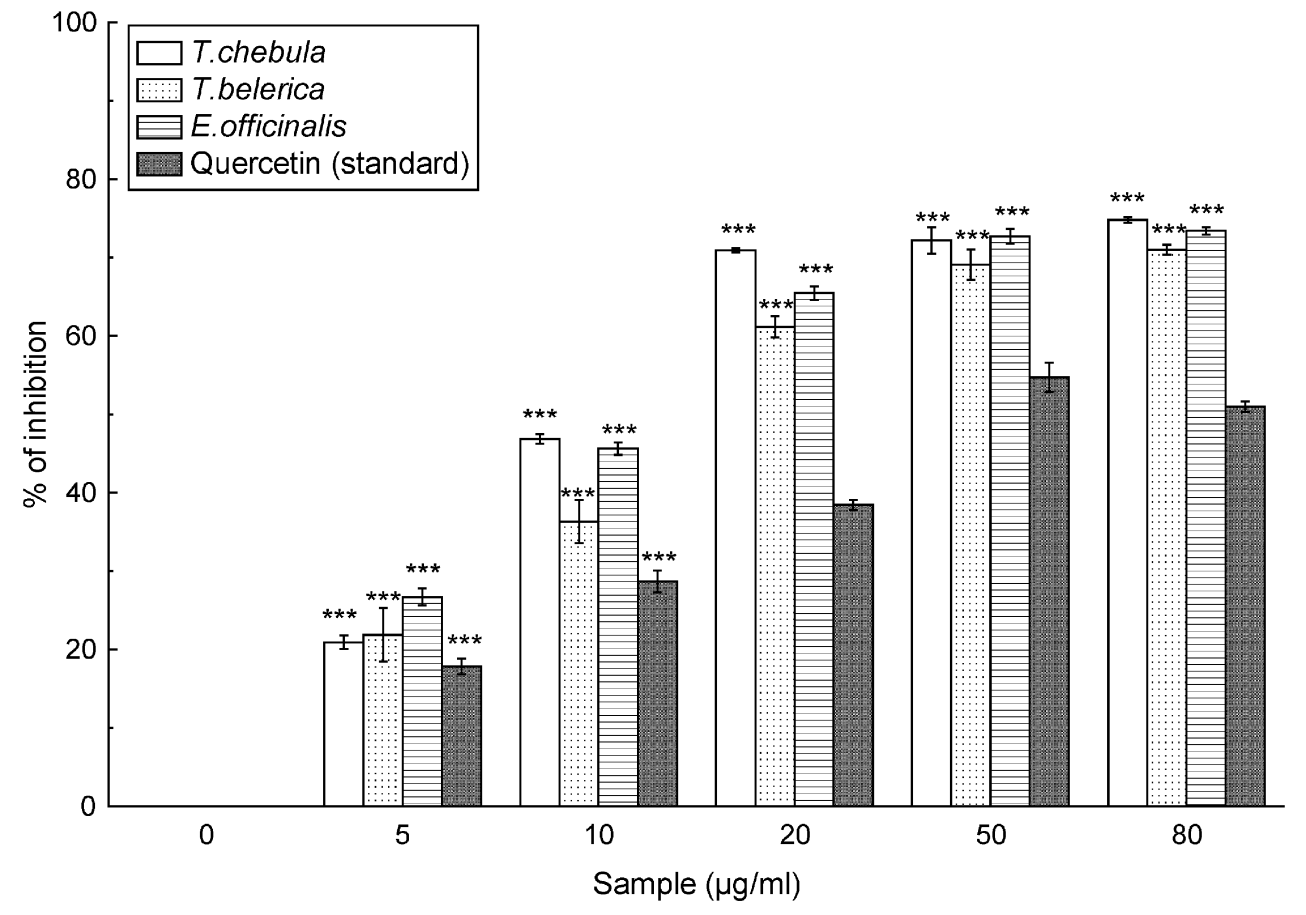
**Superoxide radical scavenging assay**. Scavenging effect of *T. chebula*, *T. belerica *and *E. officinalis *plant extracts and standard quercetin on superoxide radical. The data represents the percentage of superoxide radical inhibition. All data are expressed as mean ± S.D. (n = 6). ***p < 0.001 vs 0 μg/ml.

Figure 4 shows the abilities of the fruit extracts and the reference compound quercetin to quench superoxide radicals in the PMS-NADH reaction mixture. The IC_50 _values (Table 2) of the fruit extracts, in the order as mentioned above and quercetin were 13.42 ± 0.22 μg/ml, 18.18 ± 1.39 μg/ml, 13.82 ± 0.19 μg/ml and 42.06 ± 1.35 μg/ml, respectively.

### Nitric oxide radical scavenging assay

**Figure 5:**
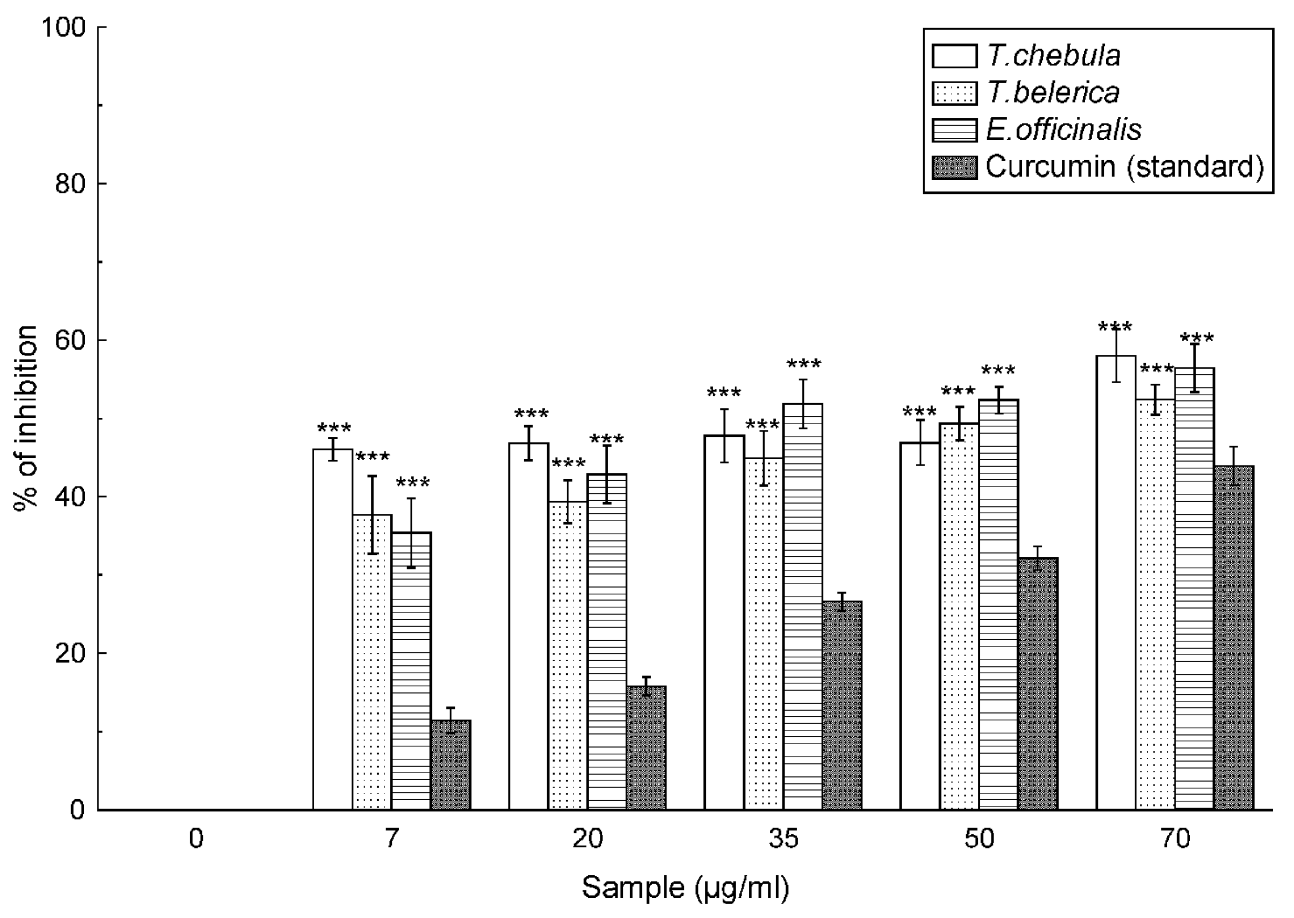
**Nitric oxide radical scavenging assay**. The nitric oxide radical scavenging activity of *T. chebula*, *T. belerica *and *E. officinalis *extracts and standard curcumin. The data represents the % of nitric oxide inhibition. Each value represents mean ± S.D. (n = 6). ***p < 0.001 vs 0 μg/ml.

As evident from Figure 5, the extracts of *T. chebula*, *T. belerica *and *E. officinalis *also caused considerable dose-dependent scavenging of nitric oxide in comparison to the reference compound curcumin, which is also reflected in their respective IC_50 _values (Table 2) of 33.28 ± 4.56 μg/ml, 40.83 ± 4.40 μg/ml, 33.89 ± 2.94 μg/ml and 90.82 ± 4.75 μg/ml for the extracts in the aforesaid order and curcumin.

### Peroxynitrite scavenging assay

**Figure 6:**
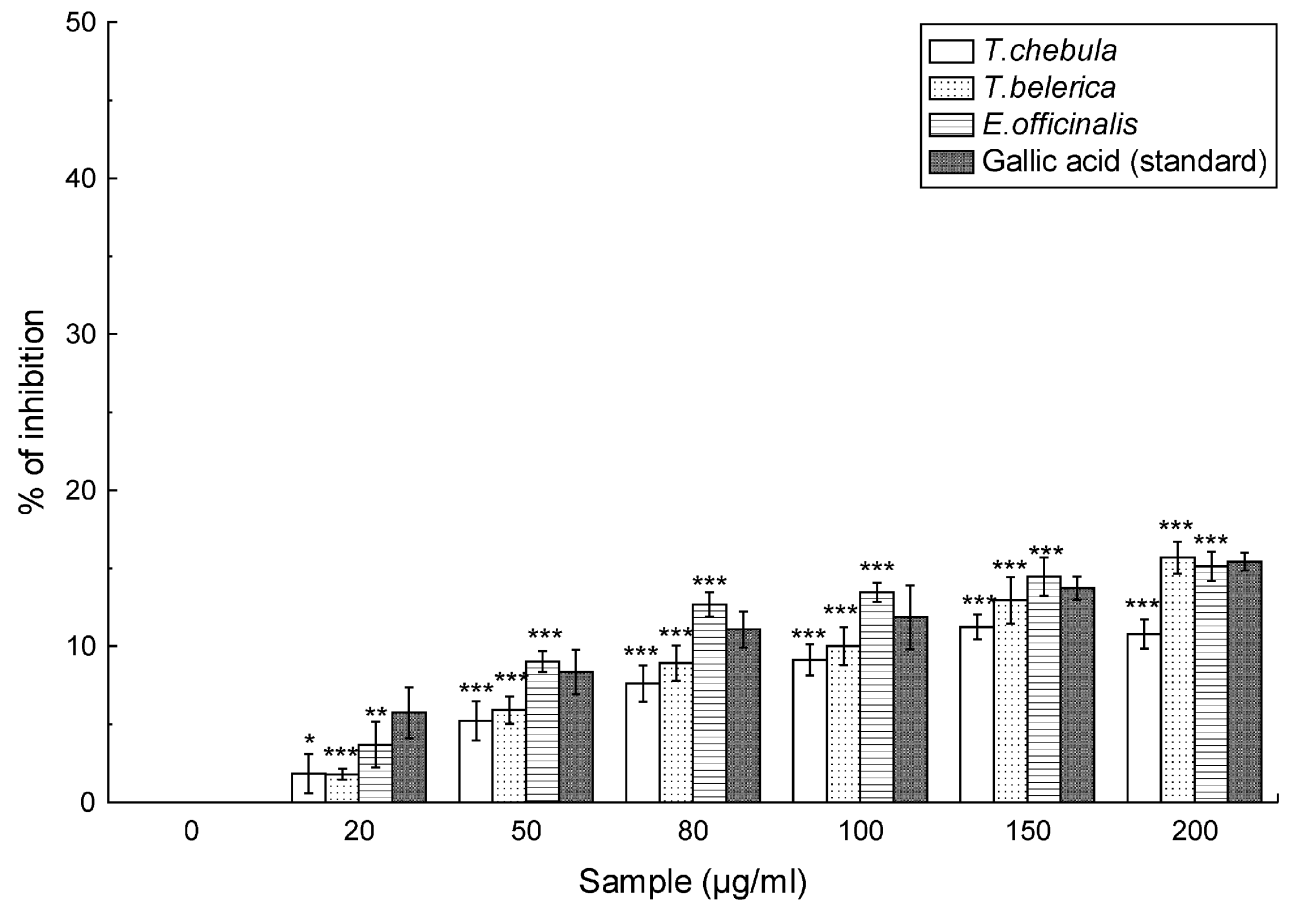
**Peroxynitrite anion scavenging assay**. The peroxynitrite anion scavenging activity of *T. chebula*, *T. belerica *and *E. officinalis *plant extracts and standard gallic acid. Each value represents mean ± S.D. (n = 6). *p < 0.05, **p < 0.01 and ***p < 0.001 vs 0 μg/ml.

Figure 6 shows the peroxynitrite scavenging activity of the fruit extracts in a concentration dependent manner. The calculated IC_50 _values for *T. chebula*, *T. belerica *and *E. officinalis *were 1.27 ± 0.07 mg/ml, 0.99 ± 0.10 mg/ml and 0.83 ± 0.03 mg/ml, repectively in comparison to that of the reference compound gallic acid (IC_50 _= 0.88 ± 0.06 mg/ml) (Table 2) indicating that the samples are not as potent scavenger of peroxynitrite as gallic acid, except *E. officinalis*.

### Hydrogen peroxide scavenging assay

Hydrogen peroxide scavenging activity of the extracts of *T. chebula*, *T. belerica *and *E. officinalis *showed no substantial result compared to the standard sodium pyruvate (IC_50 _= 3.24 ± 0.30 mg/ml) and the IC_50 _values of the same were found to be much higher than can be represented. So, no figure or IC_50 _values were provided.

### Singlet oxygen scavenging assay

**Figure 7:**
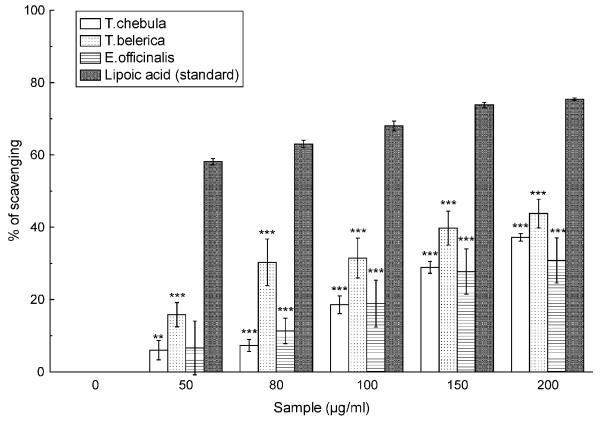
**Singlet oxygen scavenging assay**. Effect of *T. chebula*, *T. belerica *and *E. officinalis *plant extracts and standard lipoic acid on the scavenging of singlet oxygen. The results are mean ± S.D. of six parallel measurements. **p < 0.01 and ***p < 0.001 vs 0 μg/ml.

The *T. chebula*, *T. belerica *and *E. officinalis *extracts also showed a moderate dose-dependent scavenging effect of singlet oxygen species with IC_50 _values (Table 2) of 424.50 ± 24.70 μg/ml, 233.12 ± 48.68 μg/ml and 490.42 ± 159.59 μg/ml, respectively (Figure 7). Lipoic acid was used as a reference compound and 46.15 ± 1.16 μg/ml lipoic acid was needed for 50% inhibition.

### Hypochlorous acid scavenging assay

**Figure 8:**
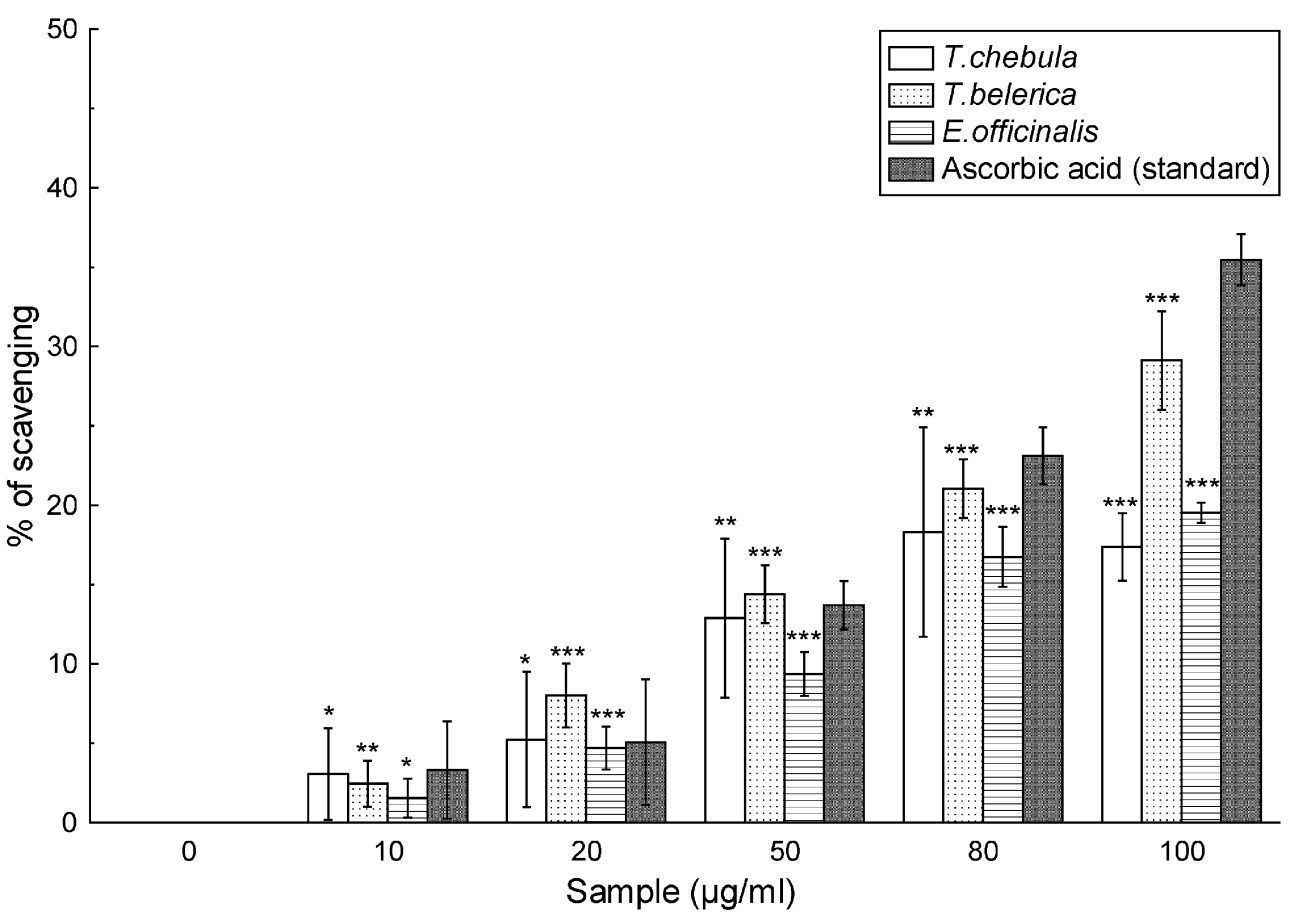
**HOCl scavenging assay**. Hypochlorous acid scavenging activity of *T. chebula*, *T. belerica *and *E. officinalis *plant extracts and standard ascorbic acid. All data are expressed as mean ± S.D. (n = 6). **p < 0.01 and ***p < 0.001 vs 0 μg/ml.

Figure 8 shows how effectively the *T. chebula*, *T. belerica *and *E. officinalis *extracts dose-dependently scavenge hypochlorous acid compared to that of ascorbic acid. The 50% inhibition concentration values of the extracts in the above order (IC_50 _= 433.60 ± 15.45 μg/ml, 271.51 ± 13.70 μg/ml, 420.58 ± 31.97 μg/ml) and ascorbic acid (IC_50 _= 235.96 ± 5.75 μg/ml) as seen in Table 2 also corroborates the data.

### Reducing power assay

**Figure 9:**
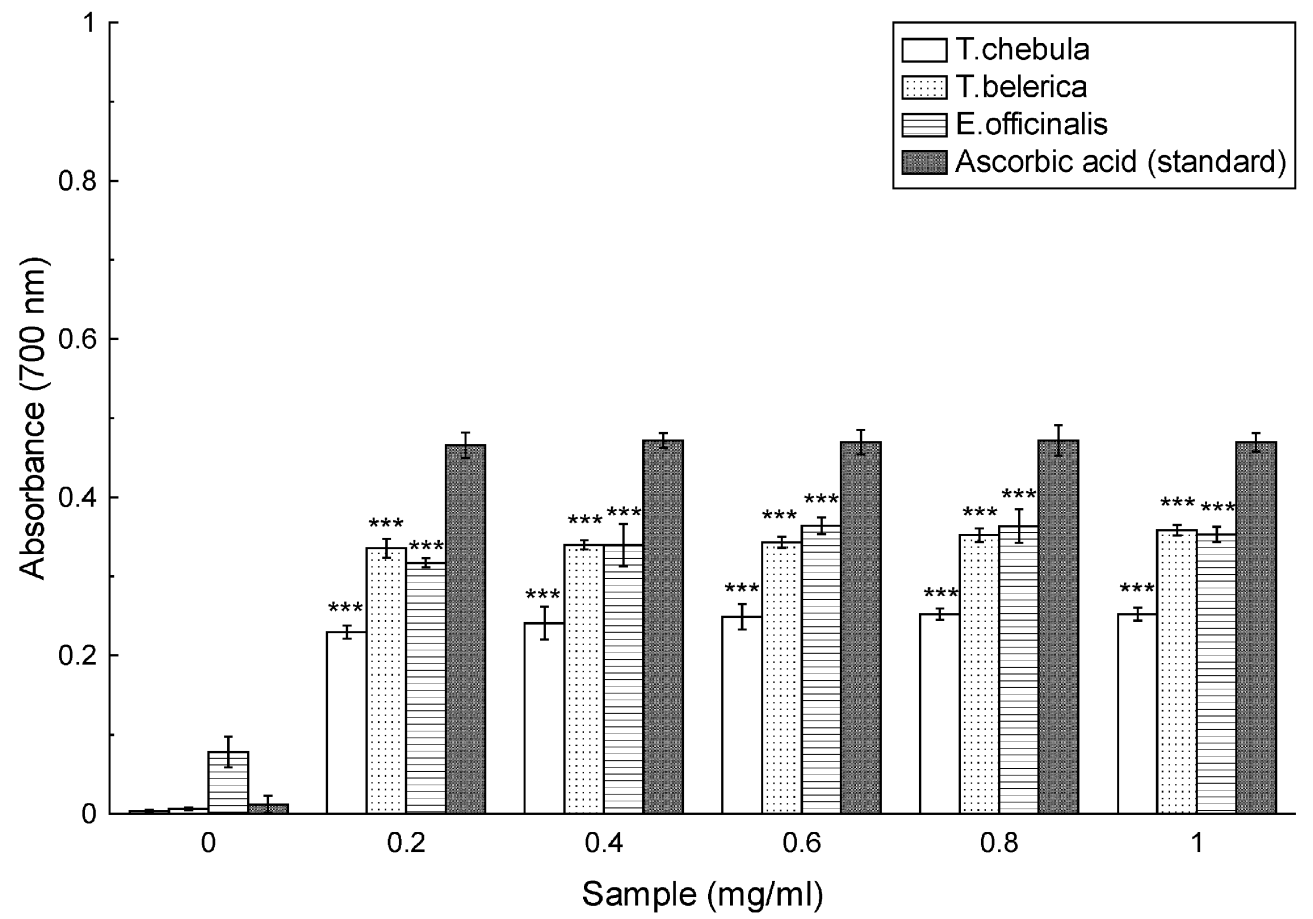
**Reducing power assay**. The reductive ability of *T. chebula*, *T. belerica *and *E. officinalis *extracts and standard Ascorbic acid. The absorbance (A_700_) was plotted against concentration of sample. Each value represents mean ± S.D. (n = 6). *** *p *< 0.001 vs 0 mg/ml.

As illustrated in Figure 9, Fe^3+ ^to Fe^2+ ^transformation in the presence of *T. chebula*, *T. belerica *and *E. officinalis *extracts and reference compound ascorbic acid was performed to measure the reductive capability. Throughout the concentration range (0.0-1.0 mg/ml), the fruits extracts and the standard showed nearly the same trend in their reductive capability, although all the extracts exhibiting lower activity than the standard.

### Determination of total phenolic, flavonoid and ascorbic acid content

The phenolic and flavonoid compounds along with the subsequent ascorbic acid contents of the extracts may contribute directly to antioxidative action. The total phenolic content of 70% methanolic extracts of *T. chebula*, *T. belerica *and *E. officinalis *were 127.60 ± 0.001 mg/ml, 133.00 ± 0.003 mg/ml and 215.60 ± 0.004 mg/ml gallic acid equivalent per 100 mg fruit extract, respectively, whereas the flavonoid contents were 219.30 ± 0.01 mg/ml, 138.30 ± 0.01 mg/ml, 176.00 ± 0.01 mg/ml quercetin per 100 mg fruit extract, following the above order. In case of the ascorbic acid content determination, 46.74 ± 1.10 mg/ml, 45.25 ± 0.75 mg/ml and 71.08 ± 1.63 mg/ml ascorbic acid were found to be present in 100 mg fruit extracts of *T. chebula*, *T. belerica *and *E. officinalis*, respectively.

### Correlation between the total phenolic or flavonoid contents with the antioxidant activity

**Figure 10:**
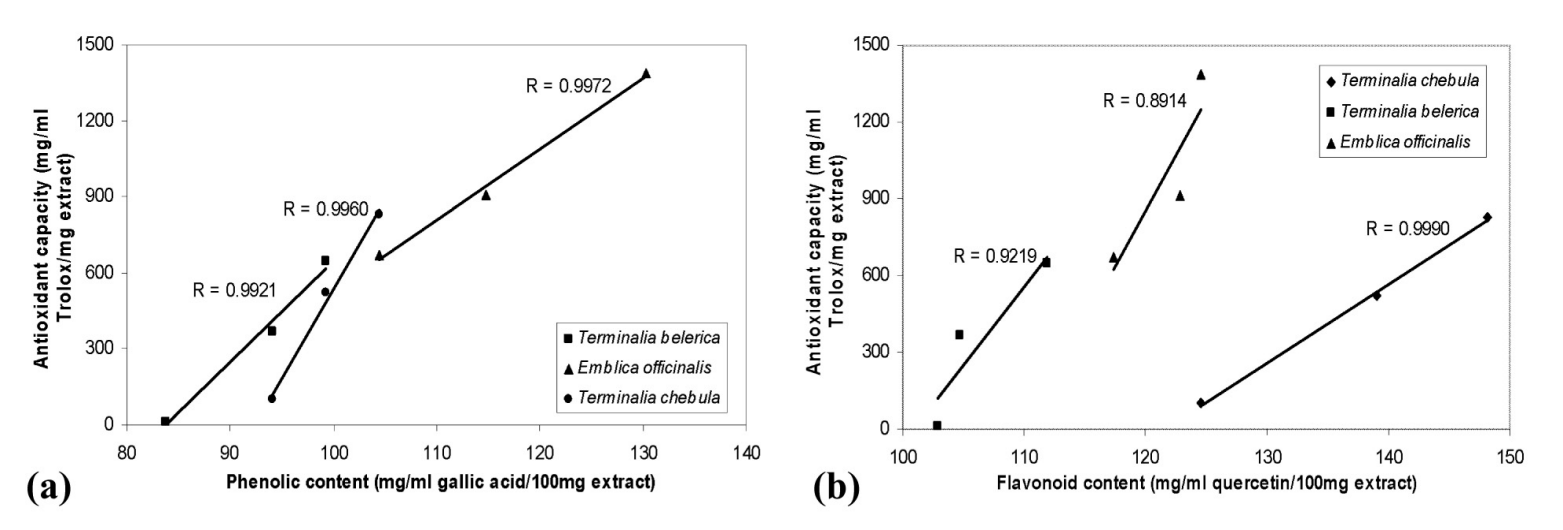
**Correlation of antioxidant activity with phenolic and flavonoid contents**. The relationship between (a) total phenolic content or (b) total flavonoid content in individual fruit and their antioxidant capacity. The correlation analyses were described as linear correlation coefficient (R). The differences were considered statistically significant if *p *< 0.05.

As showed in Figure 10(a), the total phenolic content of *E. officinalis *significantly correlated with antioxidant activity (*R *= 0.9972, *p *< 0.05), whereas the correlation coefficients for *T. chebula *and *T. belerica *were found to be greater than 0.9 (*R *= 0.9960 and *R *= 0.9921 respectively with *p *> 0.05) which proved that the phenolic contents of these plants highly attributed their antioxidant activity. The correlation coefficient of *T. chebula *for flavonoid contents with its antioxidant capacity was highly significant (*R *= 0.9990, *p *< 0.05), whereas the flavonoid contents of *T. belerica *and *E. officinalis *were highly (*R *= 0.9219, *p *> 0.05) and reasonably (*R *= 0.8914, *p *> 0.05) correlated with their antioxidant activity (Figure 10(b)). In general, the results showed that the total phenolic and flavonoid content in individual fruits was highly correlated with antioxidant activity.

### Effect on Enzyme Activity

In order to investigate whether these antioxidant activities of fruit extracts are mediated by an increase in antioxidant enzymes, the activities of SOD, CAT, GST and GSH (Table 3) were measured individually for the three plants through oral administration in mice. The activity of SOD is significantly enhanced by all three fruit extracts in a dose dependent manner. With the increasing doses of the fruit extracts, the CAT activity was also increased for each plant, in comparison to the control, as is evident from the results. The results also showed that there was a considerable dose-dependent effect of the fruit extracts in the gradual enhancement of the GST activity in the treated mice than the control ones. Similarly, we find a gradual increase in the GSH activity procured by the fruit extracts with their increasing concentrations for the dose treated mice compared to the control.

**Table 3:** Effect of the methanolic extract of the fruits of *Terminalia chebula*, *Terminalia belerica *and *Emblica officinalis *on the activities of antioxidant enzymes and reduced glutathione content in liver of normal mice

		**Tested Parameters**			
		
**Tested Samples**		**SOD (U/mg protein)**	**CAT (U/mg protein)**	**GST (U/mg protein)**	**GSH (μg/mg protein)**
		
Control	Group I	1.28 ± 0.35	0.29 ± 0.16	0.09 ± 0.04	2.8 ± 0.7
		
*Terminalia chebula*	Group II	2.33 ± 0.24***	0.51 ± 0.22	0.11 ± 0.07	3.35 ± 0.5
	Group III	3.14 ± 0.37***	0.74 ± 0.4	0.26 ± 0.05**	3.52 ± 0.5*
	Group IV	3.75 ± 0.27***	1.08 ± 0.23*	0.37 ± 0.04***	3.51 ± 0.2
		
*Terminalia belerica*	Group V	2.61 ± 0.22***	0.43 ± 0.06	0.15 ± 0.1	3.42 ± 0.3
	Group VI	3.62 ± 0.24***	0.65 ± 0.2	0.67 ± 0.15*	4 ± 0.1
	Group VII	3.89 ± 0.15***	0.66 ± 0.1**	0.71 ± 0.3	4.04 ± 0.12
		
*Emblica officinalis*	Group VIII	2.31 ± 0.18**	0.33 ± 0.2	0.14 ± 0.2	3.04 ± 0.07
	Group IX	4.01 ± 0.17***	1.02 ± 0.15**	0.41 ± 0.15*	3.85 ± 0.2
	Group X	4.56 ± 0.18***	1.06 ± 0.1**	0.64 ± 0.1**	4.13 ± 0.1

Group I: Control group; Groups II, III and IV represent mice treated orally with the *T. chebula *extract in the doses of 10, 50 and 100 mg/kg body weight, respectively; Groups V, VI and VII contain mice treated orally with the *T. belerica *extract in a dose of 10, 50 and 100 mg/kg body weight, respectively; Groups VIII, IX and X represent mice treated orally with the *E. officinalis *extract in a dose of 10, 50 and 100 mg/kg body weight, respectively. Results are expressed as mean ± SD for six mice in each group.

*p < 0.05, **p < 0.01, ***p < 0.001 compared to control.

## Discussion

Antioxidants are compounds that prevent the oxidation of essential biological macromolecules by inhibiting the propagation of the oxidizing chain reaction. Keeping in mind the adverse effects of synthetic antioxidants, researchers have channelled their interest in isolating natural antioxidants [21] which are very effective to control the oxidative stress and hence prevent the initiation of disease propagation. Interestingly, quite a few studies on the antioxidant properties of the three plant materials, viz., *T. chebula *[22,23], *T. belerica *[24,25] and *E. officinalis *[26,27] have been done earlier. However, this study provides a definitive report about the free radical scavenging capacity of *T. chebula*, *T. belerica *and *E. officinalis*, since the antioxidant activity of a drug may depend on the free radical scavenging activity [28].

ABTS^**.+ **^is a blue colored chromophore which is reduced to ABTS on a concentration dependant manner upon addition of the fruit extract. The results are compared with trolox and the TEAC value demonstrates the extracts as a potent antioxidant, with their TEAC values following the order *T. chebula *>*E. officinalis *>*T. belerica*. The effect of the fruit extract in the scavenging assay of DPPH radical furthermore assured the fact that the extracts smoothly act as antioxidants, since the study on TEAC and DPPH scavenging can be observed as complementary to each other [29], although it followed the order *T. belerica *>*E. officinalis *>*T. chebula*.

The most detrimental of the free radicals formed in biological systems is the hydroxyl radical that causes enormous damage on biomolecules of the living cells [30]. As the extracts or standard mannitol is added to the Fenton reaction mixture the hydroxyl radicals are scavenged and thereby sugar damage can be blocked. The results, as can be found from Figure 3 and Table 2, indicate that the fruit extracts are better hydroxyl radical scavengers than standard mannitol, with *T. chebula *being the best in comparison to *T. belerica *and *E. officinalis*.

Superoxide anion is also another harmful reactive oxygen species as it damages cellular components in biological systems [31]. The ability of the fruit extracts and the reference compound quercetin to quench superoxide radicals from reaction mixture is reflected in the decrease of the absorbance at λ = 560 nm. From the results (Figure 4 & Table 2), it can be put forward that the fruit extracts are more potent scavenger of superoxide radical than the standard quercetin with a decreasing order of *T. chebula *>*E. officinalis *>*T. belerica*.

Nitric oxide radicals play important roles in various types of inflammatory conditions including juvenile diabetes, multiple sclerosis, arthritis and ulcerative colitis [32,33]. The nitric oxide generated from sodium nitroprusside reacts with oxygen to form nitrite anion that is well restrained by the extracts. The scavenging activities of the extracts and curcumin proved that the nitric oxide scavenging activity of the former is better than the latter, with *T. chebula *and *E. officinalis *showing nearly the same activity, which is better that that of *T. belerica*.

Furthermore, the lethal consequence of NO increases significantly upon reaction with superoxide radical resulting in the formation of highly reactive peroxynitrite anion (ONOO^-^), especially its protonated form, peroxynitrous acid (ONOOH). ONOO^- ^has added to the pathogenesis of diseases such as heart disease, Alzheimer's disease, and atherosclerosis [34,35]. However, as revealed in Figure 6, highly considerable results were obtained for the scavenging effects of the studied extracts, which illustrated similar result to the standard gallic acid in the order *E. officinalis *>*T. belerica *>*T. chebula*.

Hydrogen peroxide is a weak oxidizing agent and can inactivate a few enzymes directly, usually by oxidation of essential thiol (-SH) groups. It can cross cell membrane rapidly, once inside the cell, H_2_O_2 _can probably react with Fe^2+ ^and possibly Cu^2+ ^ions to form hydroxyl radical and this may be the origin of many of its toxic effects [36]. From the results, it appeared that H_2_O_2 _scavenging activity of the fruit extracts is very negligible compared to standard sodium pyruvate.

A high energy form of oxygen, singlet oxygen is generated in the skin upon UV-radiation and it induces hyperoxidation, oxygen cytotxicity and decreases the antioxidant activity [37]. The higher IC_50 _values (Table 2) of the studied extracts than the reference compound lipoic acid indicated that the extracts of the fruits of *T. chebula*, *T. belerica *and *E. officinalis*, with *T. belerica *being the most effective among them, have singlet oxygen scavenging activity but poor compared to standard lipoic acid, as also found in Figure 7.

Hypochlorous acid is another harmful ROS. At the sites of inflammation, the oxidation of Cl^- ^ions by the neutrophil enzyme myeloperoxidase results in the production of this ROS [38], which breaks down the heme prosthetic group and inactivates the antioxidant enzyme catalase. The obtained results (Figure 8) indicate that the standard ascorbic acid is a comparable scavenger to the fruit extracts (Table 2). So, it is anticipated that *T. chebula*, *T. belerica *and *E. officinalis *are efficient scavengers of HOCl, with *T. belerica *being the most effective, just like in case of singlet oxygen.

The reducing capacity of a compound may serve as a significant indicator of its potential antioxidant activity. However, the activity of antioxidants has been attributed to various mechanisms such as prevention of chain initiation, decomposition of peroxides, reducing capacity and radical scavenging [39]. As shown in figure 9, the reducing power of the fruit extracts were compared with standard ascorbic acid and it was found that reducing capacity of the fruit extracts were although not better than standard, yet showed considerable activity with *E. officinalis *as the best among the three studied extracts.

The results indicate that the fruit extracts contain significant amount of flavonoids and phenolic content, in the order *T. belerica *>*E. officinalis *>*T. chebula *and *E. officinalis *>*T. belerica *>*T. chebula *for the flavonoid and phenolic contents, respectively. Both of these compounds have good antioxidant potential and their effects on human nutrition and health are considerable. The mechanism of action of flavonoids is through scavenging or chelating process [40]. Phenolic contents are also very important plant constituents because of their scavenging ability due to their hydroxyl groups [41]. Moreover, ascorbic acid acting as a chain breaking antioxidant impairs with the formation of free radicals in the process of formation of intracellular substances throughout the body, including collagen, bone matrix and tooth dentine [42]. From the results, the trend for the ascorbic acid content was found to be *E. officinalis *>*T. chebula *>*T. belerica*.

We also observed that treating mice with total extracts of medicinal plants increased the activity of all antioxidant enzymes examined, including SOD, CAT, GST and GSH. These enzymes are modulated in various diseases by free radical attack, thus maintaining the balance between the rates of radical generation and scavenging. It is of particular interest to note that SOD catalyzes the breakdown of O_2_^. ^to O_2 _and H_2_O_2_, and thus prevents the formation of OH^.^, and thereby, has been implicated as an essential defense against the potential oxygen toxicity. SOD catalyzes the breakdown of endogenous cytotoxic superoxide radicals to H_2_O_2 _which is further degraded by CAT. Thus, they play a crucial role in maintaining the physiological levels of O_2 _and H_2_O_2_. GSH, in conjunction with GST, has a basic role in cellular defense against deleterious free radicals and other oxidant species [43]. GST catalyzes the conjugation of thiol group of glutathione to electrophilic substrates, and thereby detoxifies endogenous compounds such as peroxidized lipids [44]. The present study supports the antioxidant potency of the fruit extract as evidenced by the increased level of these antioxidant systems in extract treated mice.

## Conclusions

The results from various free radical scavenging systems revealed that all the fruit extracts were individually strong antioxidants, with some varying scavenging activities for different ROS at different magnitudes of potency. Furthermore, evaluation of *in vivo *antioxidant activity of these fruit extracts has also provided interesting results that might be beneficial for the pharmacological use of these plants in clinical trials. The wide use of these fruits in the Indian indigenous system of medicine as anti-inflammatory and antihepatotoxic may be in part due to their antioxidant potency. Further, the isolation of the compounds responsible for the antioxidant activity has to be taken up which may result in modern drugs from these plants. Also the studies on antioxidant activity of the well known *Ayurvedic *formulation, Triphala, a mixture of these fruits, should be carried out and that is in progress.

## Competing interests

The authors declare that they have no competing interests.

## Authors' contributions

BH and RS both have performed the study such as extract preparation, antioxidant evaluation and animal experiments. They also have been involved in analysis of data and preparation of manuscript. SB has helped in the acquisition and statistical analysis of data. NM has been involved in study design, revising the manuscript and final approval of manuscript for submission. All authors have read and approved the final manuscript.

## Pre-publication history

The pre-publication history for this paper can be accessed here:

http://www.biomedcentral.com/1472-6882/10/20/prepub
